# An avoidable crisis

**DOI:** 10.1186/s12954-022-00680-y

**Published:** 2022-08-29

**Authors:** Catriona Matheson, Roy Robertson

**Affiliations:** 1 University of Stirling, Stirling, Scotland, UK; 2 University of Edinburgh, Edinburgh, Scotland, UK

**Keywords:** Drug policy, Political control, Overdose prevention, Benzodiazepine, Research

## Abstract

In Scotland drug policy and consequently the progress of evidence-based treatment options has been struggling for many years. Political inaction is brought about by a complex chain of legal and operational obstructions with local authorities deferring to national Government which in turn is paralysed by international convention. Scotland represents a case study demonstrating the adverse consequences of management by non medical requirements rather than implementation of a clinically proven progressive policy. The difficulty of translating theory and evidence into practice is acknowledged but suggestions are made for pragmatic and humanitarian initiatives.

## Introduction

Drug problems in Scotland share similarities with those in most European and North American countries but have demonstrated particular, and sometimes individual, difficulties over the last few decades. A series of public health crises have been associated with injecting drug use in Scotland over several decades which have alerted clinicians to a culture of drug use giving rise to problems. These include blood borne virus infection, contaminated materials and a rate of drug related deaths exceeding those of most European countries including its UK neighbour England [1–5]. It is worth exploring and trying to explain the unique features of past drug policy and clinical interventions, and to propose a better way of working, if that is possible.

There is little disagreement that drug-related damage present a gobal problem, which seems to expand and diversify year by year. In the introduction, the United Nations Drug Report of 2021 draws attention to the global scale of the drug problem and outlines the enormity of the impact of drug use.

*Drug use killed almost half a million people in 2019, while drug use disorders resulted in 18 million years of healthy life lost, mostly due to opioids. Serious and often lethal illnesses are more common among drug users, particularly those who inject drugs, many of whom are living with HIV and hepatitis C. The illicit drug trade also continues to hold back economic and social development, while disproportionately impacting the most vulnerable and marginalized, and it constitutes a fundamental threat to security and stability in some parts of the world* [6]

National and regional reports continue year by year to highlight similar patterns of collateral damage, drug-related deaths and blood-borne virus transmission [7–9].

The cost to economies is considerable and covers almost all government departments. In the UK the recent independent review by Dame Carol Black estimated the cost of drug use to UK society to be £19 billion, twice the cost of the market itself. [9]. The negative health and subsequent political impact grow with every year [10].

In responding to this set of interlinked crises it is perhaps not surprising that national policy often focuses on clinical and social consequences of drug damage and as a result reacts to reports such as those reflecting increased numbers of cases of deaths and other forms of harm associated with drugs.

There is a further problem of gathering evidence and translating this into advocacy and policy. The problems of gathering evidence in this area might be compared to other groups of marginalised people. Theory of Oakley and Harding and others draws attention, in the case of gender, to the need to empower oppressed groups in order to improve their situation [11, 12]. This resonates strongly with the need to provide evidence sympathetic to the views and needs of people who use drugs.

### Scotland’s drug problem.

Nowhere have problems been more evident over several decades than Scotland where drug-related issues seem to increase year after year. A rapid rise in numbers of people using heroin occurred in the early 1980s. Distinctly different from most other UK centres the majority of this new wave of young people involved were injecting rather than smoking the drug. Over the next 20 years clusters of cases of viral and bacterial infections were recorded including HIV, hepatitis B and C, anthrax, clostridium and botulism [2–5]. Community care and hospital departments experienced a new phenomenon of a rising tide of overdoses, sudden deaths and attendances with skin and organ infections caused by contaminated injected materials [13]. Unfamiliar diagnoses such as cellulitic skin lesions and septic foci in organs were added to differential diagnoses in hospital departments. Most recently drug-related death reports have become emblematic of the drug problem in Scotland [14].

Clinical services were initially unprepared for the complexity of cases and struggled to come to terms with the novelty of managing a problem with a collision of legal, medical and social needs in a, then young, vulnerable, population and their families and communities.

The impact of opiates and other illegal drugs on the health and social care systems have allowed many positive and some ground breaking developments. Scotland has been excellent at recording and reporting epidemics of HIV, hepatitis B and C, and other clusters of bacterial infections [2–5]. Robust systems of reporting and recording the epidemiology of the epidemics of BBVs and drug deaths have been instrumental in driving change. Good reporting may have something to do with the outlying numbers when compared to countries with less well developed monitoring. As an early adopter of needle and syringe provision this systematic approach has been maintained, evaluated, and developed over four decades [15, 16]. Primary care, community pharmacy and specialist services have managed a complex and, at times, difficult caseload and have been innovative in establishing shared care pathways of management in those communities most damaged by drug problems [17]. However, contractual arrangements negotiated between governing bodies and government have restricted the potential for this at a national level with innovation often limited to local areas. In line with national guidelines [18], Scotland has pioneered the community role out of naloxone and has greatly expanded this under the drug death taskforce strategic plan [19]. Research efforts and national reporting systems have been a model of excellence envied by colleagues in the European Monitoring Centre, Lisbon (personal communication).

These interventions and an awareness in community and specialist services of the enormity of the harms which arise for various reasons associated with drug use have highlighted the “drug problem” in Scotland and continue to stress budgets for research and clinical practice and to challenge policy makers. Political policy may have a direct or indirect influence in generating or sustaining drug-related harms. This is complex and not always obvious and there are many examples, not least the relatively poor funding of the clinical and academic sectors.

### Policy response in recent years

Almost inevitably politicians have found it difficult to develop policy pathways that are acceptable to government and electorate requirements. Resulting drug policy responses have led to a short-term focus on current single issues. The most obvious example is the concentration on rising numbers of cases of drug related deaths rather than an investigation into underlying causes. Establishing a “mission” to address this symptom of a bigger problem runs the risk of missing fundamental issues. Governments, including serial Scottish ones, have struggled to respond quickly to emerging difficulties and have eventually, often grudgingly, endorsed changes such as injecting equipment provision and opiate agonist treatments. Guidelines and national policy documents remain guarded and revert to conservative dogmas rather than responding to the evidence, which should drive change. [20, 21].

A good example of a problem, which, intuitively, requires an urgent response but in many ways is a symptom of a more systemic problem, is the record breaking total of deaths attributable to controlled drugs.

Drug-related deaths have been described as a public health emergency and in doing so have been characterised as an independent crisis. Responses include Government apologies, establishment of a taskforce with specific pathways designed to mitigate the personal, and political impact of the damaging effect of drugs and renewed activity in the treatment sector [22].

Not surprisingly inquiry into drug deaths reveals a complex set of social, economic and clinical interactions and a complex range of interventions which can be more pronounced in specific groups, such as women [1, 23] The taskforce initiated a strategic, evidence-based, plan with the expectation that Ministers would approve and deliver.

An alternative approach to a short-term allocation of resources is a more demanding and deeper radical restructuring of health and social care and addressing inequalities of opportunity or resource [7]. However, such fundamental change risks political derision and may take time to deliver long-term meaningful change.

### A more complete view of the drug problem

A focus on deaths, blood-borne viruses, or any other complication of drug use demands an understanding of the underlying cause.

In the case of drug deaths, a clear definition of a terminal event linked to a drug, its pharmacology and their individual’s physiological status is required before numbers can be calculated. Establishing a list of cases of death from the effects of drugs requires a single or a clear set of inclusion criteria. At present the diagnosis of a drug-related death in Scotland requires the presence of a controlled substance to be present in the body at the time of death. This has the advantage of simplicity but raises certain questions. Even this apparently easy definition becomes complicated when exclusions and exceptions must be made.

Examples of this case definition inadequacy are easy to find. A sudden loss of consciousness and subsequent demise from a single injection or consumption of a strong opiate in an otherwise reasonably well person is not hard to attribute directly to death by overdose. Death in a multimorbid individual, however, with multiple organ failure at the end of an illness with a drug-related cancer or tissue damage is less easily seen as a drug death. In addition, if death from a cause related to historic drug use, such as hepatitis C and hepatocellular cancer where there is no controlled drug present, is less clearly a drug death and not included in current totals. Degenhardt and others have estimated that for every recorded drug death there are another two arising from drugs but not qualifying for the current inclusion criteria [8].

A number of categories of what some might think should be counted, as "drug-related" deaths do not come within the scope of the definition because the underlying cause of death was not coded to one of the required ICD10 codes. Examples of deaths, which are not counted, for this reason are:

- Deaths coded to mental and behavioural disorders due to the use of volatile substances.
- Deaths from AIDS where the risk factor was believed to be the sharing of needles.
- Deaths from drowning, falls, road traffic and other accidents which occurred under the influence of drugs; and
- Deaths due to assault by a person who was under the influence of drugs, or as a result of being involved in drug-related criminal activities.

Other deaths that are excluded from the statistics include:

- Deaths coded to drug misuse where the direct cause of death was secondary infections or later complications of drug use. The statistics therefore exclude deaths from:
- Secondary infections such as clostridium or anthrax infection resulting from the injection of contaminated drugs;
- Conditions which could be regarded as later complications of drug use, such as bronchopneumonia, lobar pneumonia, bilateral pneumonia, septicaemia or organ failure where drug misuse was not specified as the direct and immediate cause of death (even though it may have damaged greatly the person's health over the years—so reference to, for example, ‘chronic’ or ‘long-term’ drug misuse does not necessarily mean that it was the direct and immediate cause of death).

*Source* Taken from the National Records of Scotland 2021 report [22].

Altering definitions of drug-related deaths are complex and potentially hazardous. The current definitions are based on a consensus agreed by national committees, the Home Office Advisory Committee in the case of the UK) [24]. Alteration would require a similar examination of the evidence base and consultation exercise but, in our view, would clarify the extent of the problems of drug-related harms. In the alcohol field studies of “attributable fractions” of deaths in sectors such as violence, trauma, vascular disease etc. has shown that it is possible to widen the scope, and therefore understanding of the impact of drugs on mortality Figures. [25].

At present interpretation of the cause of death is based on expert opinion of pathologists carrying out the post mortem examination in consultation with forensic toxicology results. This is subject to individual variation and local policy as well as political pressure.

### Missed opportunities and three possible policy changes that might substantially alter the landscape

We present three evidence informed policy changes that would ensure future policy and practice is sufficiently robust to address previously missed opportunities. We do recognise that policy decisions can be evidence informed rather than entirely evidence-based. However, in making these suggestions we also recommend the academic and evidence generating field lobbies harder for evidence being more central to decision making to avoid unnecessary harm in the future.

1. Improving and expanding the drug deaths definition to include a larger number of causes of death by drugs.
  *Justification* There could be several ways to include the larger caseload attributable to drug causes. For example, scrutinising death certificates to find and include diagnoses consistent with drug aetiology could significantly change numbers. Similarly, the removal of cases where the presence of a controlled drug, in the judgement of the pathologist or certifying doctor, had little to do with the death would decrease the caseload. Policy based on deaths at the end of a chain of events therefore requires a better insight into the caseload and the circumstances before and at the time of death. This could aid the justification of holistic care and pathways into primary and secondary care and the broader responsibilities of the NHS and professional bodies to be part of this system instead of annexing to specialist addiction services.
2. A unitary policy of minimising the harm from drug use by implementing policies designed to make drug harms less likely. As an example, the establishment of a heroin assisted treatment room in a single site in central Glasgow was an urgent response to a crisis of HIV transmission. In addition there should be at least one, or possibly two, safer injecting facilities in each specialist centre to complement existing injecting equipment provision and medication assisted treatment. This could be accommodated in the existing secondary care centres and may not require enormous additional investment.
  *Justification*A strong evidence base exists for provision of new sterile injecting equipment, medication assisted opiate agonist treatment and safer injecting facilities for people currently using inadequate or public spaces to use drugs.
  The binary presentation of abstinence versus harm reduction has been unhelpful in focussing resources and guiding policy.
3. A programme of clinical and social research into drug-related problems covering clinical problems and behavioural outcomes.
  *Justification* Most observers of the research base or clinical services recognise the failure of previous strategies, which promise change, but deliver more of the same. Governments rarely acknowledge this circular and unprogressive policy landscape impasse. All are beset by poor implementation and almost no delivery plans or substantial investment in good quality research.

An example of the need for further research is clear from recent policy decisions in Scotland. On an appointment of a new ‘Drugs Minister’ in Scotland a huge investment in residential rehabilitation was announced, a policy which has no evidence based in reducing drug deaths, albeit a potentially beneficial option for some, clinically suitable, people. This announcement appears to be more aimed at silencing political opponents calling for more residential rehabilitation than reducing drug-related deaths. A government appointed specialist group even noted:

*From the Group’s own discussion of the evidence base it was acknowledged that there remains a dearth of research into residential rehabilitation and recovery outcomes in Scotland. It was recognised that many questions remain unanswered and require further consideration*. [26].

Putting the cart before the horse in this way is not unusual in the drug policy world. Lack of recognition of the intergenerational vulnerabilities prevent strategic thinking about the nature of relapse and recovery [27, 28] and the absence of fundamental research obstructs the development of realistic guidelines. An example of the latter is the confusion over the long-term effects of benzodiazepines on cognitive function. Inadequate research investment leaves us, after 50 years of use of these drugs, to fail to identify the real risks [29–33].

Possible solutions to all aspects of drug problems from supply, education, treatment and research are missing due to lack of leadership or disinvestment by Governments, the academic and university sector, the NHS and other care providers.

## Conclusion

There are few other areas of public health and social policy that are led so much by political expediency rather than research evidence and which is presented by politicians who depend upon selected reports rather than peer reviewed research and often blatantly ignore evidence if it does not fit their political aims.

The politicisation of drug policy has contributed to the crisis of drug harms and nowhere is this more evident than in Scotland.

While we understand the difficulty of translating theory and evidence into the real world of politicians and political reality [34] it is in an attempt to be supportive rather than just critical that we believe would reduce further unnecessary harm.

We propose some clear policy and practice change that might align Scotland with most European countries and deliver a humanitarian and clinically realistic, evidence-based path to progress.

There is considerable evidence that a more liberal, humanitarian and inclusive approach can change outcomes [35–38]. Reports from Europe, Australia and N America draw attention to the benefits of heron assisted treatment, safer injecting facilities, and needle and syringe provision on health but also on local and community understanding of risks [39–45]. The collection and interpretation of evidence must have the same level of investment and follow the same robust approaches applied in other areas of health care.

## Data Availability

Data used is from clinical experience or published papers and reports in the public domain.
